# Exploring the landscape of physical activity and neuroplasticity research: a comprehensive bibliometric review

**DOI:** 10.3389/fspor.2025.1593690

**Published:** 2025-09-26

**Authors:** Yeting Zhang, Huan Ma, Kang Zhang, Yan Fu

**Affiliations:** ^1^ College of Aviation Physical Education, Civil Aviation Flight University of China, Guanghan, China; ^2^ College of Education Science, Sichuan Normal University, Chengdu, Sichuan, China; ^3^ College of Physical Education, Sichuan Normal University, Chengdu, Sichuan, China; ^4^ College of Physical Education, Southwest Minzu University, Chengdu, Sichuan, China

**Keywords:** exercise, brain plasticity, bibliometric analysis, cognitive function, research hotspots, development trends

## Abstract

Neuroplasticity, the nervous system's ability to adapt structurally and functionally to environmental changes, is essential for learning, memory, recovery, and overall brain health. Physical activity is recognized as an effective intervention to promote neuroplasticity, enhancing cognitive function and neural health. However, research on the overall landscape of “physical activity and neuroplasticity” is limited. This study used bibliometric analysis to elucidate the development status and evolution of the knowledge structure in this field. Data from the Web of Science Core Collection database covering literature published from January 1, 2005, to December 31, 2024, were analyzed using software such as Excel, Scimago Graphica, VOSviewer, and CiteSpace. A total of 1,854 publications meeting the selection criteria were identified, showing a gradual increase in annual publications from 2005 to 2019 and a significant surge starting from 2020. The United States ranked first in publications and citations, followed by China and Canada. The University of California, Los Angeles, and the University of Illinois had the highest number of publications, with the latter leading in citation impact. Neuroscience was the journal with the most publications and citations. Research hotspots included the mechanisms of how physical activity enhances neuroplasticity and optimal intervention modalities, with keywords like “rehabilitation” and “cognition” frequently appearing. Future research should focus on clinical validation of multimodal interventions in the elderly, optimization of experimental designs in animal models, and exploration of mechanisms in neurodegenerative diseases, contributing to the translation of research findings from basic research to clinical applications, ultimately promoting brain health and cognitive function across the lifespan.

## 1 Introduction

Neuroplasticity is a fundamental property of the nervous system, representing its ability to structurally and functionally adapt to internal and external environmental changes (1). It encompasses synaptic plasticity, which involves the enhancement or weakening of synaptic efficacy, neurogenesis, the generation of new neurons, and the dynamic reorganization of neural networks (2).This plasticity is particularly evident during early development and persists throughout the lifespan, although its magnitude typically diminishes with age (3, 4). Neuroplasticity plays a crucial role in normal cognitive functions, including learning and memory. It also has significant implications for disease rehabilitation and cognitive enhancement. For example, in neurodegenerative diseases, the ability to modulate neuroplasticity may offer a potential therapeutic approach to slow down or reverse cognitive decline (5, 6).

Physical activity has emerged as a powerful modulator of neuroplasticity. An increasing number of studies have demonstrated that exercise can enhance neuroplasticity through multiple mechanisms. Aerobic exercise, for instance, has been shown to increase the volume of the hippocampus, a brain region crucial for memory, and boost the expression of brain derived neurotrophic factor (BDNF) (7). BDNF is a key molecule in promoting synaptic plasticity and neurogenesis. Moreover, physical activity can improve cerebral blood flow, stimulate the release of other neurotrophic factors, and regulate neurotransmitter levels, all of which contribute to optimizing the functional connectivity of neural networks (8).

Despite the increasing research attention on the relationship between physical activity and neuroplasticity, there is a lack of comprehensive bibliometric analyses that objectively evaluate the overall landscape of this research area. This gap in knowledge hinders our understanding of the development trajectory, prevalent research topics, and future directions in the field of how physical activity impacts neuroplasticity. Therefore, this study is the first to utilize bibliometric methods to systematically analyze the research trends within the domains of physical activity and neuroplasticity. It aims to offer scholars a clear understanding of the progress and significant findings in this field, and to provide guidance for further research on leveraging physical activity to modulate neuroplasticity, ultimately contributing to improved cognitive function and neural health.

## 2 Methods

### 2.1 Literature search and selection strategy

**Figure 1:**
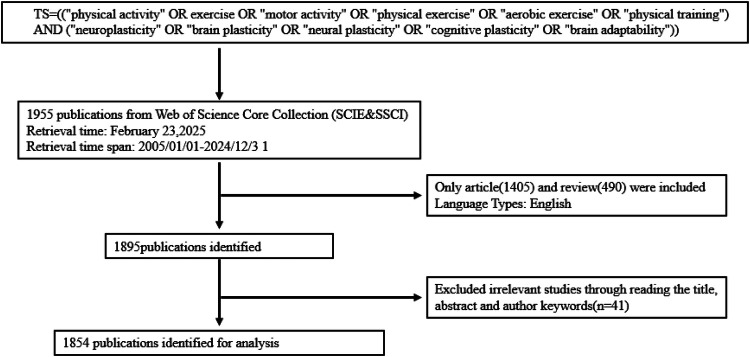
Flow chart of the literature screen.

We searched the Web of Science Core Collection database, covering the period from January 1, 2005, to December 31, 2024. The search terms were TS = ((“physical activity” OR exercise OR “motor activity” OR “physical exercise” OR “aerobic exercise” OR “physical training”) AND (“neuroplasticity” OR “brain plasticity” OR “neuroplasticity” OR “cognitive plasticity” OR “brain adaptability”)). Inclusion criteria limited publication types to “Article” and “Review”; language was restricted to English. Exclusion criteria encompassed Editorial Material, Book Chapters, Correction, Meeting Abstract, Proceeding Paper, Early Access, and Letter. After screening, we selected “Export Records to Plain Text File.” Two authors independently conducted literature screening and data extraction to minimize subjective bias. For any disputed papers, discussions with a third author were held to reach consensus (Figure 1).

### 2.2 Bibliometric analysis

We employed Excel, Scimago Graphica, an online bibliometric analysis platform, VOSviewer (v1.6.20), and CiteSpace (v.6.4.R1) for bibliometric and visualization analysis. Yearly publication volume was statistically analyzed using Excel, which allowed us to clearly observe the annual changes in the number of publications in the field of physical activity and neuroplasticity. The online bibliometric platform provided comprehensive insights into inter-country collaboration, presenting detailed information on co-authorship relationships among different countries. It also offered annual publication statistics for high-output nations, enabling us to understand the contributions of various countries over time. VOSviewer was utilized to conduct in-depth collaborative network analysis among countries, institutions, and authors. For countries, it visually represented the density of publications and citation counts, and revealed the strength of collaborative relationships through co-authorship analysis. In terms of institutions, VOSviewer helped us visualize the density maps based on publication and citation counts, and identify the collaborative networks among highly productive institutions. Regarding authors, it presented the collaborative networks among prolific authors, facilitating the understanding of their cooperation patterns. CiteSpace was applied for clustering and citation burst analysis. It was used to identify clusters of cited references, categorizing the literature into distinct themes such as aging brain, high intensity interval training, and BDNF concentration. Through citation burst analysis, CiteSpace determined the references and keywords that experienced significant citation surges over time, highlighting the emerging research trends and important works in the field.

## 3 Results

### 3.1 Analysis of annual publications

**Figure 2:**
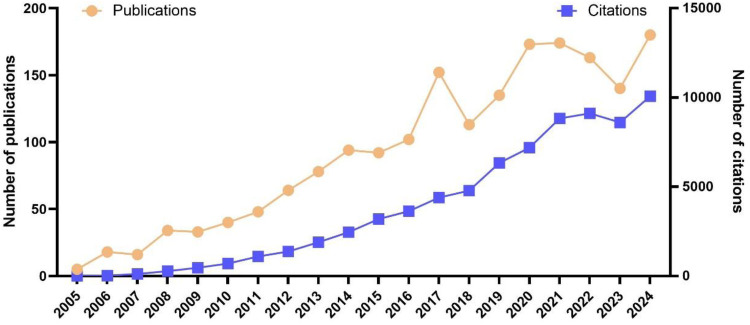
Analysis of annual academic production.

Between January 1, 2005, and December 31, 2024, a total of 1,854 publications that satisfied the specified selection criteria were identified. This cohort comprised 1,373 original research articles and 481 review articles, as depicted in Figure 1. From 2005 to 2019, there was a gradual upward trend in the annual number of publications. In 2005, the publication count in this field was a mere five articles, which increased to 135 articles by 2019. Although the growth during this period was steady, it was relatively slow, indicating the initial phase of development in this area of research. However, starting from 2020, there was a marked increase in the annual number of publications. In 2020, the number of publications surged to 173, slightly increased to 174 in 2021, and although there was a decline in 2022 and 2023 to 163 and 140 publications respectively, it rebounded to 180 in 2024. This trend reflects a growing scholarly interest in the field, potentially linked to the emergence of related research topics or an increase in societal demand. It is important to note that this observed growth in publications related to physical activity and neuroplasticity occurs against the backdrop of a general global increase in scientific production and collaboration networks in recent years, largely due to technological advances. While this broader context does not undermine the significance of the growth in our specific field, it provides a wider perspective for understanding the overall trend. The change in citation frequency further corroborates this observation. Citations increased significantly from two in 2005 to 10,073 in 2024, indicating an expanding academic impact of the research outcomes. Particularly after 2017, the rate of increase in citation frequency has notably accelerated (Figure 2).

### 3.2 Analysis of countries

**Figure 3:**
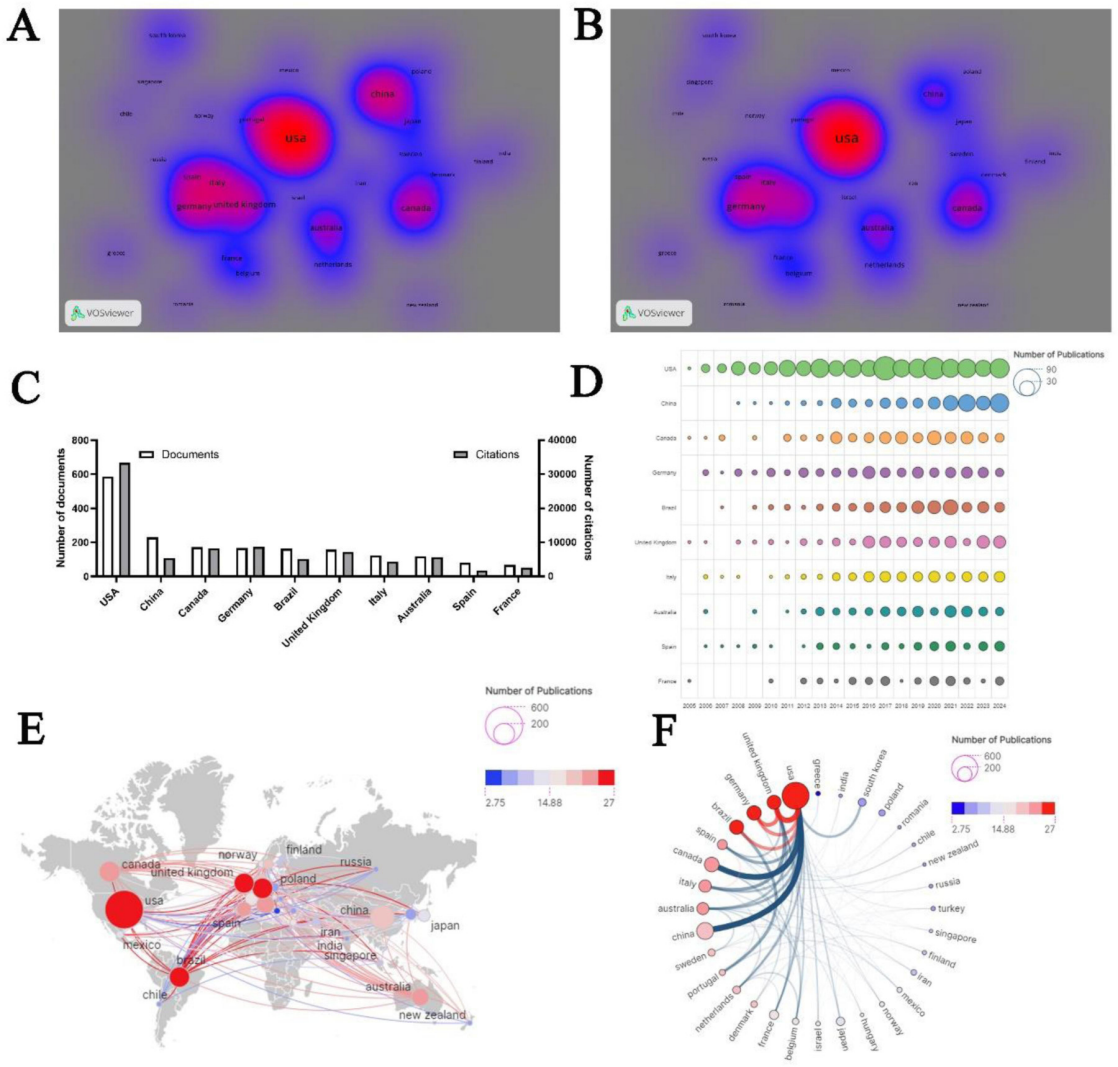
Country analysis. **(A)** Density visualizations based on document counts. **(B)** Density visualizations based on citation counts. **(C)** Top 10 countries by publication output. **(D)** Annual publication trends of the top 10 countries. **(E)** Geographic distribution of collaborative networks among countries/regions. **(F)** Map of national collaboration intensity.

A total of 76 countries and regions have contributed to this field. The volume of publications and their citation counts can be illustrated using density visualization (Figures 3A,B). As depicted in Figure 3C, among the top 10 countries ranked by the number of publications, the United States leads with 593 papers, followed by China (*n* = 205) and Canada (*n* = 172). In terms of total citation counts, the United States, Germany, and Canada rank first, second, and third, respectively, with citation counts of 33,400, 8,649, and 8,267 (Figure 3D). Figure 3D also presents the annual publication trends of the top 10 research productive countries over the past two decades, with China exhibiting the most remarkable annual growth. Co-authorship analysis among these countries reveals that the United States, Canada, and Germany have the highest levels of collaboration, with total link strengths (TLS) of 267, 123, and 116, respectively. Notably, the United States has the strongest collaborative network with countries and regions in Europe and the Americas (Figures 3E,F).

### 3.3 Analysis of organizations

**Figure 4:**
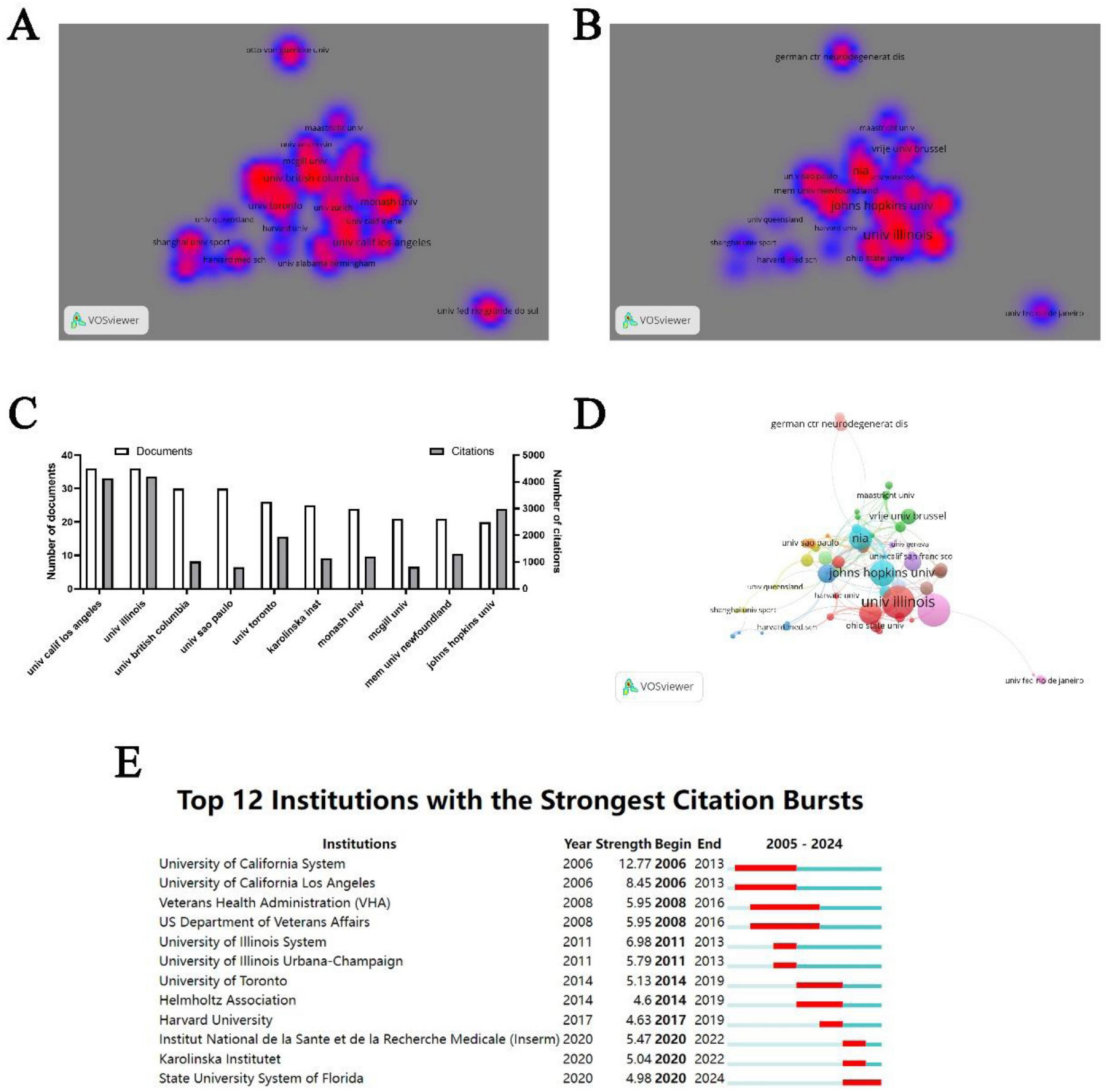
Institutional analysis. **(A)** Density visualization map based on publication counts. **(B)** Density visualization map based on citation counts. **(C)** Top 10 institutions ranked by publication output. **(D)** Collaboration network visualization among institutions. **(E)** Citation burst analysis of institutions.

A total of 2,430 institutions have contributed to the literature on physical activity and neuroplasticity. Among these, 63 institutions have published more than 10 papers each. Co-authorship analysis was employed to visualize these 63 institutions, generating density maps based on publication and citation counts (Figures 4A,B). Among the top 10 institutions by publication volume, the University of California, Los Angeles, and the University of Illinois led with 36 publications each, followed by the University of British Columbia and the University of São Paulo, each with 30 publications. In terms of citation impact, the University of Illinois took the lead with 4,206 citations (Figure 4C). Subsequent network visualizations revealed that these highly productive institutions maintain a close-knit collaboration network, particularly the University of British Columbia (TLS = 35), the University of Toronto (TLS = 33), and McGill University (TLS = 33), indicating robust inter-institutional relationships. Additionally, Beijing Normal University and Kyung Hee University have not established collaborative ties with other institutions (TLS = 0) (Figure 4D). A burst citation analysis identified 12 universities that have experienced citation bursts, with the University of California System exhibiting the most significant burst (intensity = 12.77). The State University System of Florida is currently in a phase of citation burst (Figure 4E).

### 3.4 Analysis of authors

**Figure 5:**
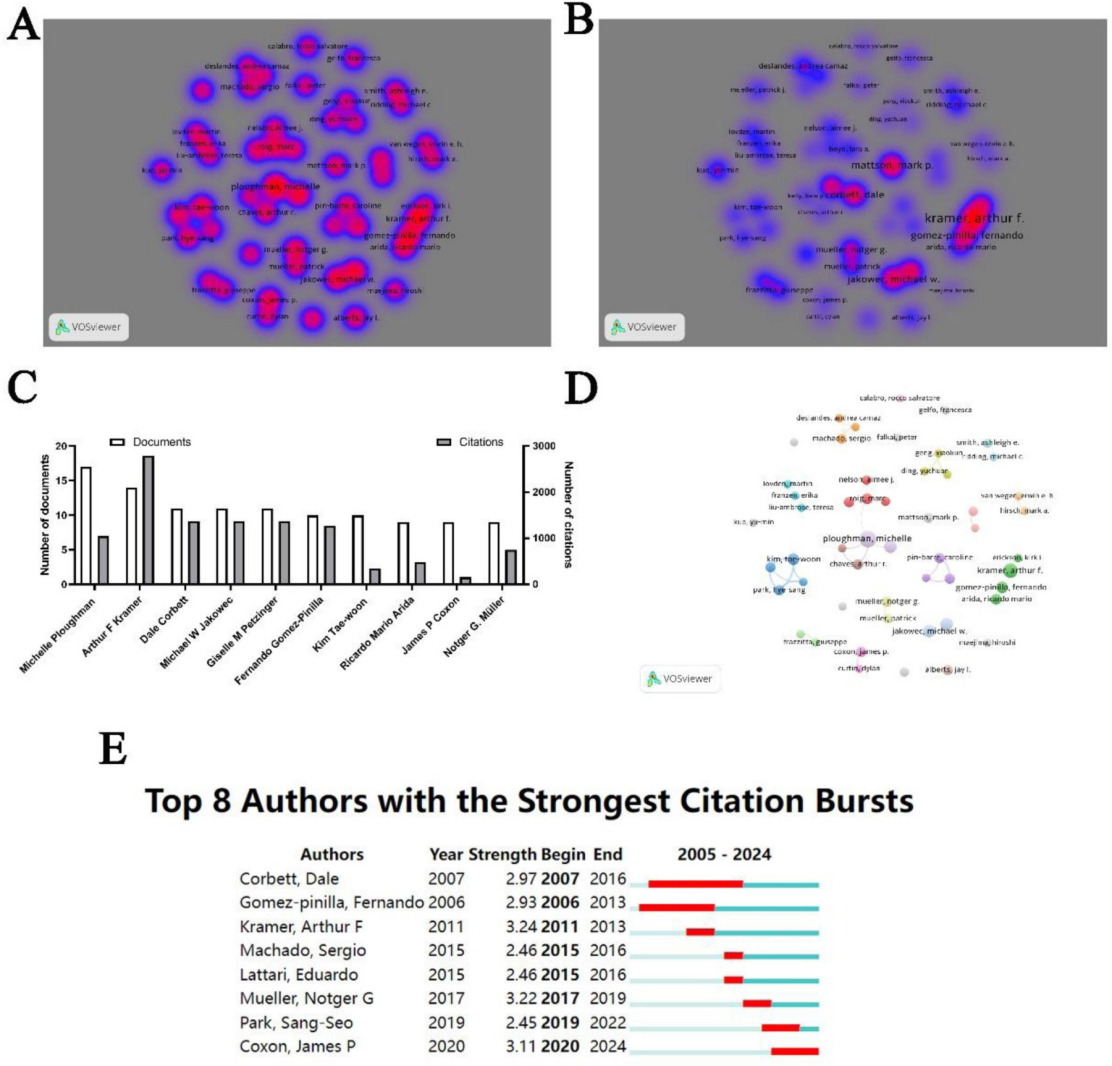
Author analysis. **(A)** Density map visualizing publication output. **(B)** Density map visualizing citation impact. **(C)** Ranking of the top 10 authors by publication count. **(D)** Network diagram depicting collaborative relationships among authors. **(E)** Analysis of citation bursts among authors.

A total of 8,915 authors have contributed to the literature in the field of physical activity and neuroplasticity, with 52 authors having published more than six papers each. An analysis of the collaborative networks among these 52 prolific authors is presented, with their productivity and citation metrics visualized using density plots (Figures 5A,B). The top ten most productive authors include Michelle Ploughman from Memorial University Newfoundland, who has authored 17 papers and garnered 1,048 citations. Following her is Arthur Kramer from the University of Illinois, who has published 14 papers and holds the highest number of citations (*n* = 2,789) (Figure 5C). Network visualizations illustrate the collaborations among these highly productive authors. Scholars in this domain have formed numerous collaborative clusters, albeit with limited inter-cluster collaboration (Figure 5D). A burst citation analysis reveals that eight authors have experienced citation bursts, with Arthur Kramer exhibiting the strongest burst (intensity = 3.24), while James Coxon from Monash University is currently in a period of heightened citation activity (Figure 5E).

### 3.5 Analysis of journals

**Figure 6:**
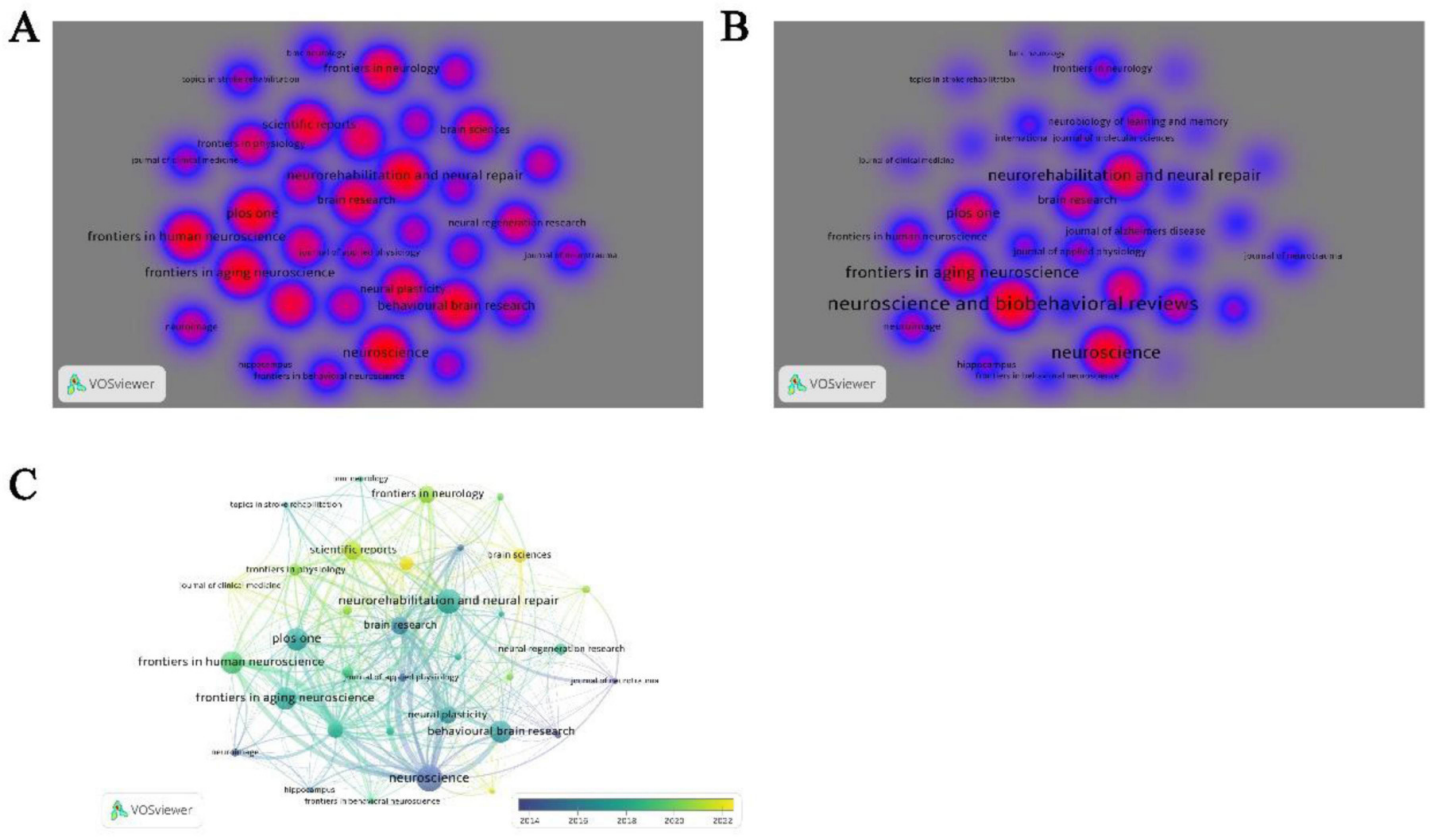
Journal analysis. **(A)** Density map of journals based on publication counts. **(B)** Density map of journals based on citation counts. **(C)** Overlay visualization of bibliographic coupling among academic journals.

A total of 563 journals has published articles on the topic of physical activity and neuroplasticity, with 35 of these journals contributing more than 10 articles each. The distribution of publications and citation counts is illustrated in density visualizations (Figures 6A,B). The journal with the highest number of publications is Neuroscience, which has published 44 articles, and it also leads in terms of citation frequency, with a total of 2,781 citations. Following closely is Neurorehabilitation and Neural Repair with 41 articles. Tied for third place are Frontiers in Aging Neuroscience, Frontiers in Human Neuroscience, and PLOS One, each with 38 articles. The top ten journals by publication volume are presented in Table 1, with Frontiers in Aging Neuroscience having the highest impact factor at 4.1. Among these, three journals are ranked in the Q1 category of the Journal Citation Reports (JCR), five in the Q2 category, and two in the Q3 category. Bibliographic coupling analysis conducted by VOSviewer on journals with more than 10 publications revealed that Neuroscience, Journal of Neurotrauma, and European Journal of Neuroscience were prominent in terms of early publication volumes. In contrast, International Journal of Molecular Sciences and Brain Sciences have shown a higher volume of recent publications (Figure 6C).

**Table 1:** Top 10 journals by publication output.

Source	Documents	Citations	Impact factor (2023)	JCR
Neuroscience	44	2,781	2.9	Q2
Neurorehabilitation and neural repair	41	2,169	3.7	Q1
Frontiers in aging neuroscience	38	2,333	4.1	Q2
Frontiers in human neuroscience	38	914	2.4	Q3
Plos one	38	1,729	2.9	Q1
Behavioural brain research	37	980	2.6	Q2
Scientific reports	31	516	3.8	Q1
Brain research	30	1,297	2.7	Q3
Frontiers in neurology	29	657	2.7	Q2
Neuroplasticity	28	1,489	3.0	Q2

### 3.6 Analysis of keywords

**Figure 7:**
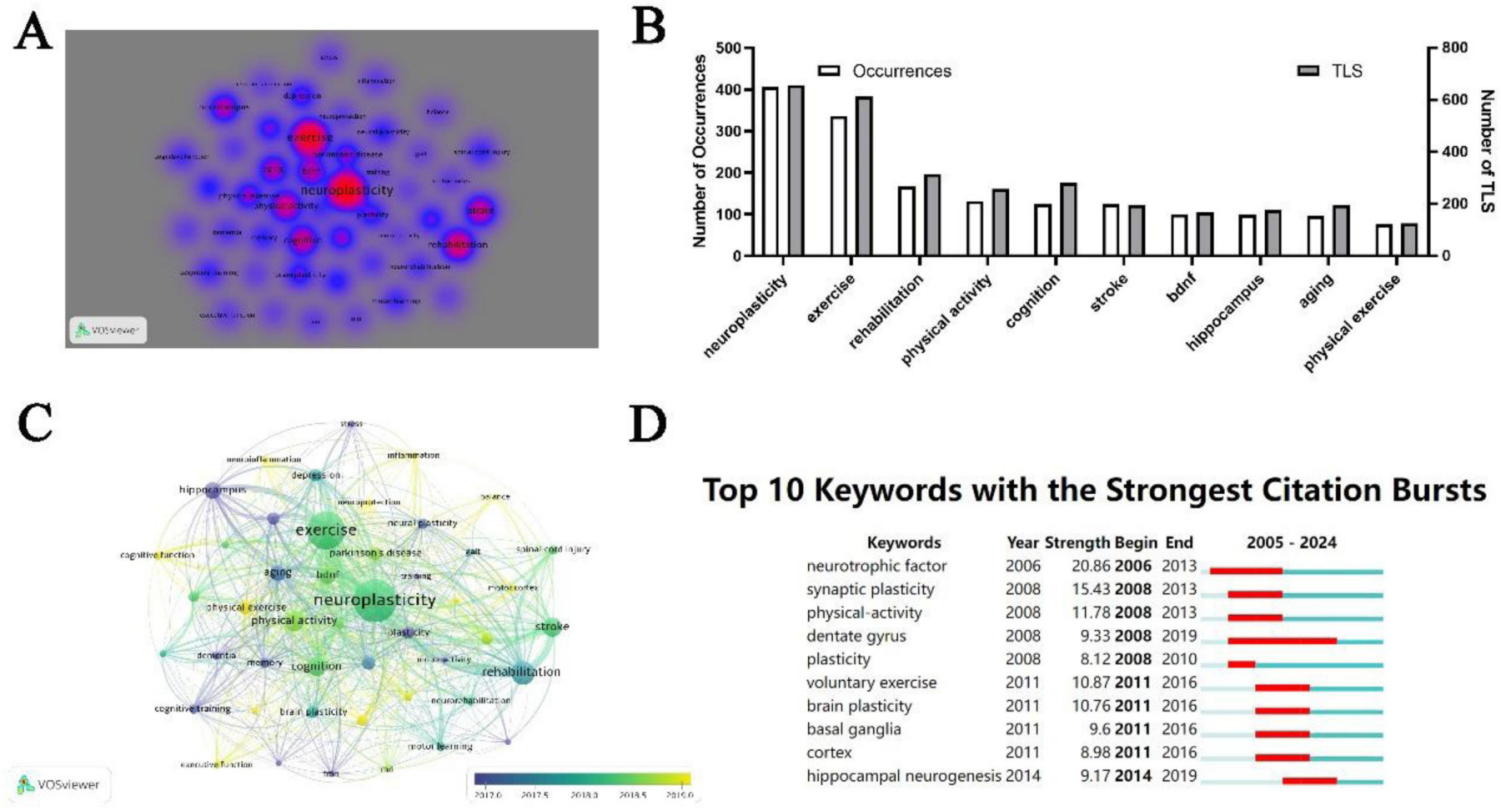
Keyword analysis. **(A)** Visualization of keyword density based on frequency of occurrence. **(B)** Top 10 keywords by frequency. **(C)** Overlay visualization of keyword trends. **(D)** Analysis of citation bursts among keywords.

**Figure 8:**
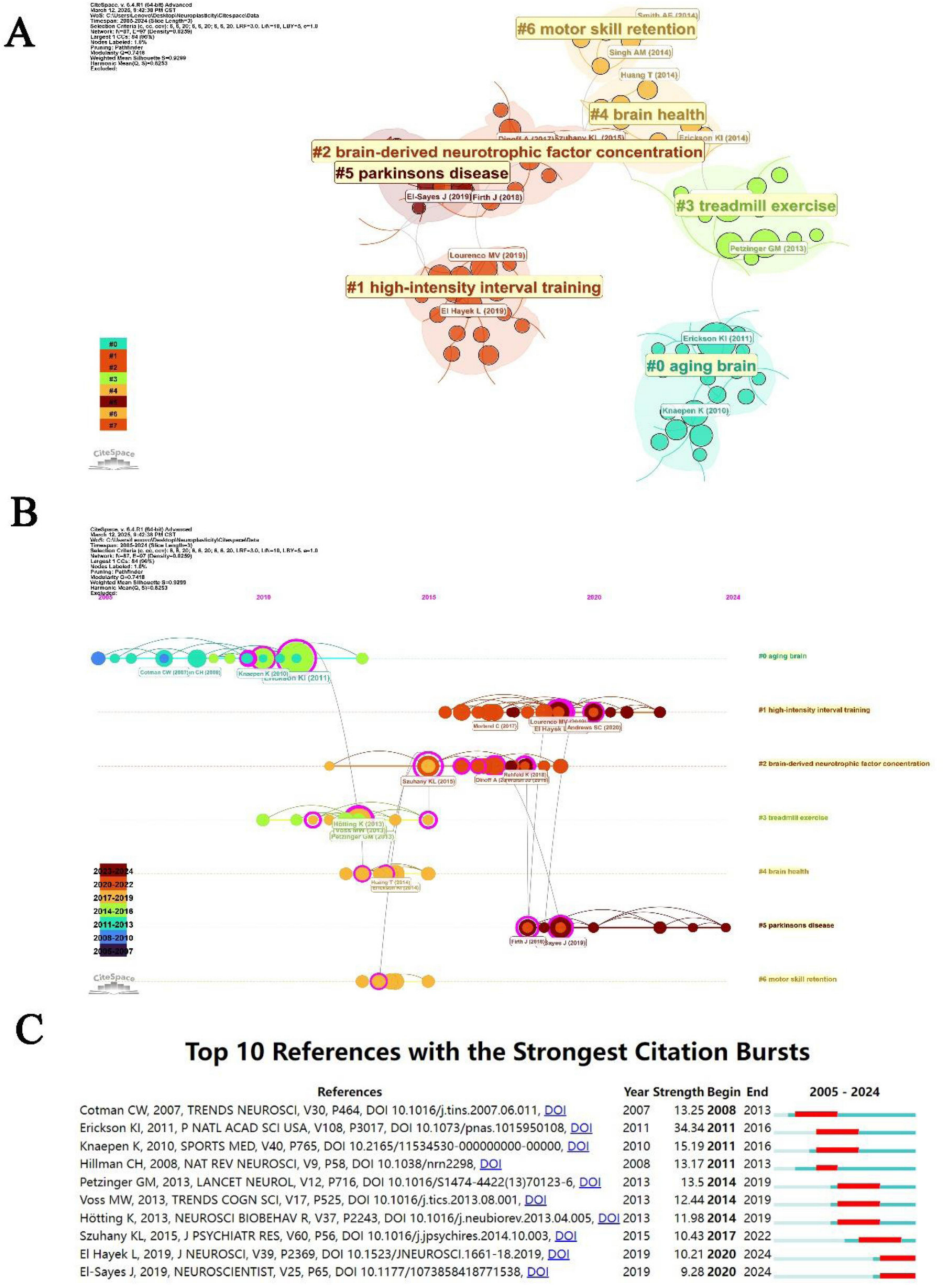
Analysis of references. **(A)** The clusters of co-cited references. **(B)** The timeline view of co-cited references. **(C)** Citation bursts analysis of references.

The objective of co-occurrence analysis is to play a pivotal role in monitoring the trajectory of scientific development by exploring predominant research directions and fields. Utilizing the VOSviewer software, we selected and analyzed keywords that appeared in no fewer than 20 publications, ultimately identifying 47 keywords (Figure 7A). The top ten keywords, ranked by frequency of occurrence, are neuroplasticity, exercise, rehabilitation, physical activity, cognition, stroke, BDNF, hippocampus, aging, and physical exercise (Figure 7B). The superimposed visualization chart indicates that keywords such as inflammation, motor cortex, aerobic exercise, executive function, and older adults have garnered increased attention in recent years (Figure 7C). Furthermore, an analysis of citation bursts among keywords revealed that a total of 58 keywords experienced citation surges. Figure 8D illustrates the top ten keywords in terms of citation bursts, with neurotrophic factor exhibiting the highest burst strength (intensity = 20.86), and dentate gyrus demonstrating the longest duration of citation burst activity (spanning from 2008 to 2019).

### 3.7 Analysis of references

Table 2 presents the top 10 most frequently cited references in the literature concerning physical activity and neuroplasticity. The article with the highest citation count is “Exercise training increases size of hippocampus and improves memory,” authored by Kirk I. Erickson et al., published in the Proceedings of the National Academy of Sciences of the United States of America in 2011, which has been cited 74 times (9). Following this is “Exercise-enhanced neuroplasticity targeting motor and cognitive circuitry in Parkinson's disease,” by G. M. Petzinger et al., published in Lancet Neurology in 2023, which has garnered 40 citations (10). Additionally, the article “Bridging animal and human models of exercise-induced brain plasticity,” by Michelle W. Voss et al., published in Trends in Cognitive Sciences in 2013, has received 37 citations (11).Co-citation analysis conducted using CiteSpace identified several clusters of cited references, encompassing seven distinct categories: aging brain, high-intensity interval training (HIIT), brain-derived neurotrophic factor (BDNF) concentration, treadmill exercise, brain health, Parkinson's disease, and motor skill retention (Figure 8A). Prior to 2015, references related to the aging brain, treadmill exercise, brain health, and motor skill retention were more prevalent. In contrast, post-2015, the focus of cited literature shifted towards HIIT, BDNF concentration, and Parkinson's disease (Figure 8B). An analysis of citation bursts among these references revealed a total of 64 bursts, with the top 10 displayed in Figure 8C. The article with the highest citation burst intensity was “Exercise training increases size of hippocampus and improves memory,” published by Kirk I. Erickson et al. in 2011 in the Proceedings of the National Academy of Sciences of the United States of America (intensity = 34.34) (9). Additionally, the articles “Lactate Mediates the Effects of Exercise on Learning and Memory through SIRT1-Dependent Activation of Hippocampal Brain-Derived Neurotrophic Factor (BDNF)” by Laurette El Hayek et al., published in 2019 in the Journal of Neuroscience (12), and “Exercise-Induced Neuroplasticity: A Mechanistic Model and Prospects for Promoting Plasticity” by Jenin El-Sayes et al., published in 2019 in The Neuroscientist (13), are currently experiencing citation bursts.

**Table 2:** Top 10 cited references by citations.

Title	Journal	First author	Year	Total citation frequency
Exercise training increases size of hippocampus and improves memory	PROCEEDINGS OF THE NATIONAL ACADEMY OF SCIENCES OF THE UNITED STATES OF AMERICA	Kirk I. Erickson	2011	74
Exercise-enhanced neuroplasticity targeting motor and cognitive circuitry in Parkinson's disease	LANCET NEUROLOGY	Giselle M Petzinger	2013	40
Bridging animal and human models of exercise-induced brain plasticity	TRENDS IN COGNITIVE SCIENCES	Michelle W Voss	2013	37
Lactate Mediates the Effects of Exercise on Learning and Memory through SIRT1-Dependent Activation of Hippocampal Brain-Derived Neurotrophic Factor (BDNF)	JOURNAL OF NEUROSCIENCE	Lauretta El Hayek	2019	35
Beneficial effects of physical exercise on neuroplasticity and cognition	NEUROSCIENCE AND BIOBEHAVIORAL REVIEWS	Kirsten Hötting	2013	34
Exercise-Induced Neuroplasticity: A Mechanistic Model and Prospects for Promoting Plasticity	NEUROSCIENTIST	Jenin El-Sayes	2019	33
Neuroplasticity — Exercise-Induced Response of Peripheral Brain-Derived Neurotrophic Factor	SPORTS MEDICINE	Knaepen K	2010	32
Exercise-linked FNDC5/irisin rescues synaptic plasticity and memory defects in Alzheimer's models	NATURE MEDICINE	Lourenco, M.V	2019	32
A meta-analytic review of the effects of exercise on brain-derived neurotrophic factor	JOURNAL OF PSYCHIATRIC RESEARCH	Kristin L Szuhany	2015	30
Intensity Matters: High-intensity Interval Exercise Enhances Motor Cortex Plasticity More Than Moderate Exercise	CEREBRAL CORTEX	Sophie C Andrews	2020	24

## 4 Discussion

The investigation into the relationship between physical activity and neuroplasticity has garnered substantial interest among researchers. As depicted in Figure 2 of our manuscript, research within this domain has generally exhibited an upward trajectory. Given the surge in the number of published articles and the persistent trend, it is crucial for researchers to grasp the prevailing research trends, focal points, and existing challenges within this field. Consequently, conducting a bibliometric analysis of the correlation between physical activity and neuroplasticity is of paramount importance. Such an analysis can provide researchers with objective data derived from previous literature, allowing them to gain a clear and comprehensive understanding of research trends, focal points, and challenges. These insights will render future research more targeted, ultimately aiding researchers in determining the relationship between physical activity and neuroplasticity and exploring its underlying mechanisms.

The United States leads in both the volume of publications and the number of citations in this field, with the majority of primary and corresponding authors of internationally published academic articles hailing from the US. Consequently, the US holds significant influence in research within this domain. China and Canada follow as the second and third most prolific publishers, respectively, while Germany and Canada rank second and third in terms of citation rates. Future research efforts should prioritize increasing citation impact and fostering international collaborations, particularly by emulating the US and strengthening research partnerships in this field. Such collaborations could substantially boost the research capabilities of participating nations. Factors such as severe demographic aging, advanced healthcare systems, and high rates of research cooperation may contribute to the significant impact these countries have in this area. Importantly, the surge in citation rates indicates that this leading influence persists. Institutions like The State University System of Florida, along with Australian author James Coxon from Monash University, have recently experienced substantial increases in citation counts.

A comprehensive statistical analysis of academic publication output has revealed a striking trend: the top ten journals consistently rank within the Q2 category or higher in the prestigious Journal Citation Reports (JCR). This trend underscores the widespread interest in research related to physical activity and neuroplasticity among esteemed academic publications. Publishing research papers in these highly regarded journals not only enhances public recognition but also stimulates further academic interest in the mechanisms by which physical activity influences neuroplasticity. Moreover, a bibliometric coupling analysis of these journals presents significant potential for researchers. By employing such analytical methods, scholars can gain insights into current publishing trends and preferences regarding articles on physical activity and neuroplasticity. This strategic approach enables researchers to make informed decisions when selecting appropriate journals for submission. Furthermore, it facilitates effective tracking of advancements in the field, ensuring that scholars remain abreast of the evolving academic discourse surrounding physical activity interventions related to neuroplasticity.

An in-depth analysis of the reference literature and target keywords has elucidated the predominant research foci and trending topics within the domain of physical activity and neuroplasticity. The findings indicate that the literature garnering substantial citations predominantly centers on the mechanisms by which physical activity enhances neuroplasticity and the optimal modalities for achieving maximal benefits. It is noteworthy that keywords such as “rehabilitation” and “cognition” frequently emerge, suggesting a profound interest in the cognitive function improvements and associated rehabilitative effects afforded by the modulation of neuroplasticity through physical activity. This pattern underscores the widespread attention garnered by the clinical applications of physical activity's impact on neuroplasticity. Recent studies have demonstrated that exercise modalities, including aerobic exercise, high-intensity interval training, and resistance training, significantly augment the expression of neurotrophic factors, thereby promoting neurogenesis, synaptic plasticity, and brain remodeling, with notable neuroprotective effects in neurological disorders such as stroke, Parkinson's disease, Alzheimer's disease, and spinal cord injury (14–16). However, the specific forms of physical activity and the methods for quantifying physical activity levels, as well as investigating their dynamic impacts on specific brain regions to further clarify the underlying mechanisms, remain an area of active research and debate. In light of these factors, research on the relationship between physical activity and neuroplasticity continues to attract sustained and intense attention from the scientific community. Future studies should focus on the clinical validation of multimodal interventions (such as various forms of physical activity, cognitive training, and social interaction) in elderly populations. This can be achieved through randomized controlled trials and the optimization of experimental designs in animal models to investigate their long-term effects on neuroplasticity and cognitive function, and to explore their mechanisms of action in a variety of neurodegenerative diseases. Ultimately, the goal is to establish a complete pipeline from basic research to clinical application, thereby facilitating the translation and application of research findings.

This CiteSpace analysis provides a comprehensive overview of the research landscape in the field of physical activity and neuroplasticity, identifying eight major clusters that reflect the evolution of the field from 2007 to 2024. The largest cluster, Aging Brain (Cluster #0), focuses on the neuroprotective effects of aerobic exercise, with Erickson et al. demonstrating increased hippocampal volume and improved cognitive function in older adults, establishing a foundational framework for exercise as a neuroprotective intervention (9). Although physical activity and training may transiently elevate BDNF levels and enhance its synthesis and utilization efficiency, there is currently no conclusive evidence to suggest that exercise can sustainably elevate baseline BDNF levels over the long term (17). Furthermore, aerobic exercise has been shown to exert positive effects on specific aspects of brain function, improving cognitive performance and behavioral outcomes in both humans and animal models (18).The High-Intensity Interval Training (HIIT) cluster (Cluster #1) highlights the molecular mechanisms by which HIIT upregulates brain-derived neurotrophic factor (BDNF) to enhance synaptic plasticity, as evidenced by El Hayek et al., who demonstrated that HIIT promotes neuroplasticity through BDNF-mediated pathways (12). Hugues et al. further explored the therapeutic potential of HIIT in neurodegenerative diseases, while Andrews et al. investigated how HIIT not only serves as an exercise intervention to enhance neuroplasticity but also improves the therapeutic potential of non-invasive brain stimulation (19, 20).The BDNF cluster (Cluster #2) explores the role of BDNF in mediating exercise-induced neuroplasticity, with Szuhany et al. providing a comprehensive review of BDNF's role in psychiatric and neurodegenerative conditions, serving as a pivotal reference for bridging basic and clinical research (21). Dinoff et al. and Walsh et al. further examined the effects of exercise intensity on BDNF expression and its implications for cognitive health (22, 23). The Treadmill Exercise cluster (Cluster #3) focuses on the effects of treadmill training on neuroplasticity, particularly in Parkinson's disease. Studies show that exercise interventions activate cognitive circuits involved in motor learning, improving behavioral functions and motor control through experience-dependent neuroplasticity (10). Voss et al. and Hötting et al. further investigated the effects of treadmill exercise on brain network connectivity and hippocampal plasticity, respectively (11, 24). These findings reveal exercise-induced synaptic and neural circuit plasticity, supporting treadmill training as a therapeutic approach for Parkinson's disease. The Brain Health cluster (Cluster #4) examines the long-term effects of physical activity on cognitive function and neuroprotection, with Erickson et al. and Voss et al. highlighting the role of exercise in preserving brain structure and function (25, 26). Ngandu et al. emphasized the importance of lifestyle interventions, including exercise, in maintaining brain health (27). The Parkinson's Disease cluster (Cluster #5) examines the role of exercise in enhancing brain connectivity and cognitive performance in patients with Parkinson's disease. El-Sayes et al. demonstrated that exercise therapy significantly increases serum levels of brain-derived neurotrophic factor (BDNF) and improves motor symptoms in Parkinson's patients (28). Langeskov-Christensen et al. emphasized that exercise, as a safe rehabilitation strategy, plays a foundational role in the management of Parkinson's disease and may have profound implications for clinical practice (29). Furthermore, Johansson et al. found that aerobic exercise enhances functional connectivity between the anterior putamen and the sensorimotor cortex, improves cognitive control, and mitigates brain atrophy, thereby stabilizing the progression of sensorimotor network dysfunction and enhancing cognitive performance in Parkinson's disease (30).The Motor Skill Retention cluster (Cluster #6) focuses on the effects of acute exercise on motor learning and memory consolidation, particularly in stroke rehabilitation, with Smith et al. (2014) and Singh et al. providing evidence that exercise enhances motor learning and memory consolidation (31, 32). Skriver et al. demonstrated that a single bout of cardiovascular exercise can enhance motor skill learning by optimizing long-term motor memory retention, however, the underlying mechanisms remain to be fully elucidated and warrant further investigation (33). The field is evolving toward a deeper understanding of molecular mechanisms, clinical translation, and interdisciplinary approaches, with future research poised to optimize personalized exercise interventions for neurodegenerative and psychiatric disorders, integrating advanced methodologies to advance our understanding of exercise-induced neuroplasticity and brain health.

A critical reflection on the field of physical activity and neuroplasticity has revealed several key issues. First, there is an imbalance between overstudied and understudied areas. While the effects of aerobic exercise on hippocampal volume and cognitive function have been well documented (9), other types of physical activity and their long-term cognitive benefits remain underexplored. Second, inconsistencies in the findings of highly cited studies, such as those on the effects of exercise on BDNF levels and neuroplasticity (12, 17, 21), highlight the need for standardized protocols and larger sample sizes to resolve discrepancies. Third, methodological flaws in key papers, including reliance on cross-sectional designs and variability in animal models and exercise interventions, limit the robustness of conclusions (18). Future research should prioritize longitudinal and randomized controlled trials, develop standardized exercise protocols, and integrate advanced neuroimaging techniques to enhance the accuracy and reliability of research findings in this field.

## 5 Limitations

There are some limitations in this study. First, due to the inherent limitations of bibliometric tools and databases, it was not feasible to integrate and analyze data from multiple databases or languages effectively. Consequently, the analysis is limited to the Web of Science Core Collection (WoSCC) database. We acknowledge that relying solely on WoSCC may have limitations, as relevant literature not indexed by this database was not included in our analysis. Nevertheless, WoSCC is widely recognized for its extensive coverage and high-quality standards in the field of academic publishing. As recent similar bibliometric studies have demonstrated, WoSCC remains a reliable source for bibliometric analysis in neuroscience research (34). We believe that the analysis based on WoSCC data can effectively capture the overall landscape and trends in this field. Secondly, this study focuses solely on English-language literature, excluding academic results in other languages, which may introduce a language bias and limit the generalizability of the findings. Third, citation counts are influenced not only by the intrinsic quality of the research but also by other factors, such as the visibility of the journal, the authors' networks, or the recency of publication. Additionally, citation practices can vary significantly across different fields and subfields, which may further distort the interpretation of citation counts as a measure of research impact. Furthermore, the literature review spans from 2005 to 2024, which may have restricted the consideration of earlier foundational studies or more recent emerging research. Consequently, there may be some omissions or biases in the data. To enhance the comprehensiveness and accuracy of future studies, it is recommended to expand data sources to include multilingual literature and a wider range of literature types, such as conference proceedings, books, and grey literature. Future studies should consider a broader range of metrics, including altmetrics and qualitative assessments, to provide a more nuanced understanding of research impact. Additionally, future research could benefit from incorporating more diverse bibliometric tools and databases to provide a more holistic view of the field.

## 6 Conclusion

In conclusion, this manuscript employs advanced bibliometric techniques to conduct a comprehensive and objective quantitative analysis of the published literature in the specialized field of physical activity and neuroplasticity. This approach not only elucidates the current research landscape and trends within this domain but also identifies significant research hotspots that have garnered substantial attention among scholars. Furthermore, it systematically addresses the prevailing challenges and gaps in the existing body of knowledge, providing a foundational reference to guide future investigations and stimulate further exploration in this critical area of study. The insights derived from this analysis hold significant implications for the direction of forthcoming research initiatives, particularly in optimizing personalized exercise interventions and advancing therapeutic strategies for neurodegenerative and psychiatric disorders through innovative applications of physical activity. By integrating interdisciplinary approaches and leveraging advanced methodologies, this research has the potential to significantly contribute to the understanding of exercise-induced neuroplasticity and its clinical applications, ultimately promoting brain health and cognitive function across the lifespan.
